# Recurrent Aseptic Meningitis Without Identifiable Pathogens: A Case Report

**DOI:** 10.7759/cureus.88161

**Published:** 2025-07-17

**Authors:** Saima Nazish, Abdulhadi J Alotaibi, Obaid M Aljarbou, Abdulaziz Omair, Faisal H Aljamea

**Affiliations:** 1 Neurology, Imam Abdulrahman Bin Faisal University, Dammam, SAU; 2 Medicine and Surgery, Vision College, Riyadh, SAU

**Keywords:** case report, diagnostic challenges, meningitis, pathogen identification, recurrent meningitis

## Abstract

Recurrent meningitis without an identifiable pathogen is an infrequent but taxing diagnostic dilemma, particularly when multiple systemic comorbidities obscure both the clinical picture and the laboratory data.

A 66-year-old man with dyslipidemia, diabetes, hypertension, ischemic cardiomyopathy, and a left ventricular thrombus experienced five meningitic episodes over six years. Each event featured holocephalic headache radiating to the neck, neck stiffness, vomiting, transient dysarthria, and gait ataxia. The cerebrospinal fluid (CSF) consistently showed lymphocytic pleocytosis (6-48 cells/mm^3^), elevated protein (75-142 mg/dL), and sterile cultures. Serial viral polymerase chain reaction (PCR) panels, bacterial and mycobacterial studies, fungal cultures, extended autoimmune screens, complement levels, and paraneoplastic panels were negative. Brain magnetic resonance imaging (MRI) revealed pachymeningeal enhancement without parenchymal lesions.

Empiric antivirals and broad-spectrum antibiotics were commenced but discontinued once repeated microbiological testing remained negative. The patient received supportive care, namely, analgesia, hydration, and close neurological monitoring, under a multidisciplinary team comprising neurology, infectious disease, and cardiology specialists. Symptoms resolved within five days; inflammatory markers and CSF indices normalized, and he was discharged with outpatient follow‑up.

When exhaustive investigations fail to reveal an infectious or systemic cause, idiopathic aseptic meningitis should be considered. Optimal care hinges on a structured diagnostic algorithm, early involvement of multiple specialties, and judicious avoidance of unnecessary antimicrobials. Documenting and sharing such cases refines clinical awareness and may eventually elucidate the still-obscure mechanisms driving idiopathic recurrent meningitis.

## Introduction

Meningitis is a severe and sudden infection of the meninges, which can be triggered by bacteria, viruses, parasites, or fungi [1]; however, sometimes, there is inflammation of the brain meninges caused by various factors, leading to negative cerebrospinal fluid (CSF) bacterial cultures known as aseptic meningitis. Occasionally, these episodes are recurrent, leading to hospitalization and diagnostic workup. Recurrent meningitis is uncommon, affecting 2-9% of meningitis cases. It presents diagnostic challenges due to its various underlying causes. The primary reasons include anatomical defects (59%), immune system deficiencies (36%), and infections around the meninges (5%); other contributing factors may include head injuries, chronic inflammation within the brain, and issues with the complement system [2,3]. Recurrent episodes of aseptic meningitis are characterized by repeated attacks of headache, fever, and meningeal signs accompanied by CSF lymphocytic pleocytosis (white blood cell (WBC) >5 cells/mm³) and sterile CSF cultures; this clinical pattern is classically referred to as Mollaret's meningitis, first described by Pierre Mollaret in 1944 [4]. These episodes of meningitis are usually caused by herpes simplex virus type 2 (HSV-2) infection, although other viral and non-viral causes have also been reported [5,6]. The causes of aseptic meningitis can be broadly classified into infectious and non-infectious origins. Despite progress in diagnostic methods, only 30-65% of cases reveal a precise cause [7]. Cases without identified causes are termed idiopathic, though recently identified conditions, such as transient headache and neurologic deficits with CSF lymphocytosis (HaNDL) syndrome and meningitis-retention syndrome, may help reduce the proportion of cases classified as idiopathic aseptic meningitis [8]. The objective of writing this case report is to discuss diagnostic challenges physicians face for these rare causes of meningitis. By integrating this knowledge into their clinical practice, clinicians will be better equipped to deliver high-quality care, improving patient outcomes and safety. We also highlighted the importance of considering the timely adaptations of available treatment options in patients with recurrent meningitis without identifiable pathogens.

## Case presentation

We present the case of a 66-year-old right-handed man with a complex medical history, including dyslipidemia, hypertension (HTN), diabetes mellitus (DM), ischemic heart disease, and left ventricular thrombus with heart failure, on apixaban 5 mg PO BID. He had a history of recurrent aseptic meningitis, 4-5 episodes in total over a six‑year period. Every time, he used to present with symptoms including headache, neck stiffness, vomiting, dysarthria, and ataxia with raised inflammatory markers and high CSF protein and white blood cell (WBC) counts with reactive atypical lymphocytes without any identifiable cause. In the first instance, antibiotics and antivirals were started. Meanwhile, we performed a workup, and the patient was discharged with normal CSF studies and inflammatory markers. Tables 1-2 show the record of the patient's CSF parameters and microbiological results over different periods.

**Table 1:** Quantitative blood and CSF findings during four admissions †: Elevated CSF glucose values reflect concurrent serum hyperglycemia at the time of lumbar puncture; adult CSF reference interval ~45-80 mg/dL. WBC: white blood cells; CRP: C‑reactive protein; ESR: erythrocyte sedimentation rate; CSF: cerebrospinal fluid; RBC: red blood cells; L: lymphocytes

Parameter	Reference range	March 2019	September 2019	June 2023	December 2023
Peripheral blood					
WBC (×10³/µL)	4.0-11.0	6.2	7.1	5.6	6.4
CRP (mg/L)	0-5	5.6	14.7	8	5.4
ESR (mm h⁻¹)	0-15	60	99	44	51
Procalcitonin (ng/mL)	<0.50 ng/mL (values ≥0.50 ng/mL raise concern for significant systemic bacterial infection)	0.13	0.11	0.14	0.44
CSF chemistry					
Protein (mg/dL)	15-45	75	132	119	85
Glucose (mg/dL)†	45-80	227	127	95	120
CSF cytology					
RBC (cells mm⁻³)	0-5	205	1	2	1
WBC (cells mm⁻³)	0-5	15 (99 % L)	22 (99 % L)	48 (82 % L)	6 (99 % L)

**Table 2:** Qualitative microbiology and molecular studies —: test not performed; WBC: white blood cell; AFB: acid-fast bacillus; PCR: polymerase chain reaction; MTBC:* Mycobacterium tuberculosis* complex; HSV: herpes simplex virus

Test	March 2019	September 2019	June 2023	December 2023
Gram stain	No organisms	Rare WBC, none seen	No organisms	No organisms
Aerobic culture (48 hours)	No growth	No growth	No growth	No growth
India ink	Negative	Negative	Negative	Negative
AFB smear/culture	Negative (6 weeks)	Negative (6 weeks)	Negative (8 weeks)	Negative (6 weeks)
*Mycobacterium *PCR	—	—	MTBC DNA not detected	—
HSV PCR	Negative	Negative	Negative	Negative
Fungal culture (4 weeks)	No growth	No growth	—	No growth
Peripheral blood culture (5 days)	No growth	No growth	No growth	No growth

This time, the patient again presented with a four-day history of a holocephalic, pressure-like headache, moderate in severity, radiating to the neck and aggravated by coughing. Associated symptoms included photophobia, neck stiffness, vomiting, unsteady gait, and generalized fatigability. Notably, there was no photophobia or fever. On examination, the patient was alert and oriented with stable vital signs. Pupils were small with sluggish light reaction; there were positive signs of meningeal irritation. Cogwheel rigidity, more pronounced in the right upper extremity, was present; rapid alternating movements were mildly slowed and irregular.** **Sensory examination was normal with pyramidal signs. Considering his medical history and the complexity of recurrent meningitis without an identifiable pathogen, comprehensive microbiological testing of the CSF was performed to rule out infectious causes. This included screening for HSV-1 and HSV-2, human immunodeficiency virus (HIV), Epstein-Barr virus (EBV), cytomegalovirus (CMV), enteroviruses, mumps, measles, atypical bacteria such as *Mycoplasma pneumoniae*, *Mycobacterium tuberculosis*, *Treponema pallidum*, and *Brucella*, as well as parasites and fungi. CSF Gram stain, aerobic cultures, and multiplex viral polymerase chain reaction (PCR) panels were obtained, and all returned negative; extended fungal culture (four‑week incubation) was also negative.** **Fungal culture, with a four-week incubation period, yielded no fungal growth. *Brucella* testing, including DNA PCR, isolation, and genetic sequencing, returned negative. Tuberculosis testing using the GeneXpert *Mycobacterium tuberculosis* (MTB)/rifampicin (RIF) assay and a six-week CSF culture showed no MTB DNA detection or growth. CSF cytology didn't reveal any malignant cells. In addition to CSF analysis, an extensive hematological, inflammatory, metabolic, and immunological workup was conducted. A broad range of non-infectious causes were considered, including systemic diseases such as Behçet's disease, Sjögren's syndrome, sarcoidosis, systemic lupus erythematosus, granulomatosis with polyangiitis, and paraneoplastic syndromes as summarized in Table 3.

**Table 3:** Expanded CSF and systemic investigations at the current admission †: CSF glucose elevated in the setting of serum hyperglycemia; adult CSF reference ~45-80 mg/dL. ANA: antinuclear antibody; dsDNA: double‑stranded DNA; SSA/SSB: Ro/La autoantibodies; ANCA: anti‑neutrophil cytoplasmic antibody; MPO: myeloperoxidase; PR3: proteinase‑3; CCP: cyclic citrullinated peptide; HIV: human immunodeficiency virus; HSV: herpes simplex virus; VZV: varicella‑zoster virus; CMV: cytomegalovirus; EBV: Epstein-Barr virus; CSF: cerebrospinal fluid; WBC: white blood cell; L: lymphocytes; AFB: acid-fast bacillus; MTB/RIF: *Mycobacterium tuberculosis*/rifampicin resistance; TSH: thyroid-stimulating hormone

Panel/assay	Reference range	Result
Autoimmune/inflammatory serology		
ANA, anti‑dsDNA, anti‑Smith	N/A	Negative
SSA/SSB, anti‑β₂‑glycoprotein	N/A	Negative
ANCA (MPO, PR3)	N/A	Negative
Rheumatoid factor, anti‑CCP	N/A	Negative
Complement C3	90-180 mg/dL	Normal
Complement C4	10-40 mg/dL	Normal
Paraneoplastic antibody panel	N/A	Negative
Infectious disease workup		
HIV, hepatitis panel	N/A	Non‑reactive
Brucella IgG/IgM, syphilis serology	N/A	Negative
Viral PCR (HSV‑1/2, VZV, CMV, EBV, enterovirus)	N/A	Negative
CSF indices (repeat tap, day 3)		
Protein	15-45 mg/dL	142 mg/dL
Glucose†	45- 80 mg/dL	138 mg/dL
WBC	0-5 cells/mm³	89 cells mm⁻³ (98 % L)
Gram stain/culture	N/A	No organisms/no growth
India ink, fungal culture	N/A	Negative/no growth
AFB smear/culture (8 weeks)	N/A	Negative
GeneXpert MTB/RIF	N/A	No MTB DNA detected
Toxicology and others		
Drug screen	N/A	Negative
TSH	0.4-4.0 mIU/L	Within reference limits
Vitamin B₁₂	200-900 pg/mL	Within reference limits
Folate (serum)	≥4.0 ng/mL	Within reference limits

**Figure 1:**
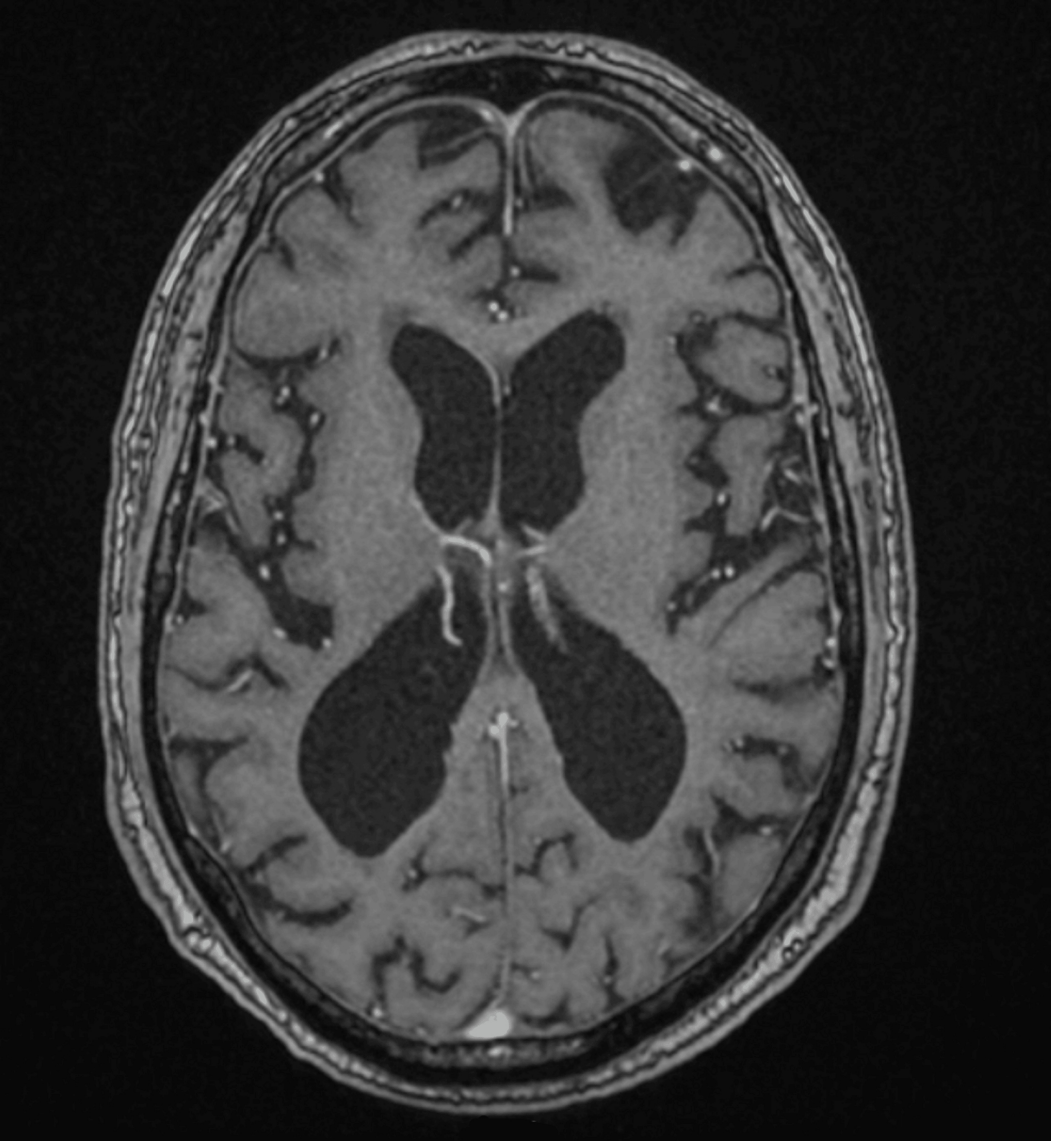
Axial T1-weighted brain MRI MRI: magnetic resonance imaging

Complement levels (C3, C4), soluble interleukin‑2 receptor, and a comprehensive toxicology drug screen were performed; all were within reference limits/negative.** **Drug-induced aseptic meningitis was also ruled out. HaNDL syndrome was also considered, but its criteria were not met. Thorough brain parenchymal and vascular imagings were performed to rule out potential inflammatory conditions, particularly vasculitis. Brain magnetic resonance imaging (MRI) showed mild ventricular enlargement, persistent pachymeningeal enhancement, and mild cerebral small vessel disease (Figure 1).

Interdisciplinary care, with multiple teams, including infectious disease, neurology, and cardiology, was involved to work together to address the patient's clinical condition. The patient's clinical presentation, lack of identifiable pathogens, and exclusion of common causes led to a working diagnosis of aseptic meningitis. After excluding both infections and non-infectious causes of aseptic meningitis, ultimately, an idiopathic aseptic meningitis was considered. The patient was managed with a combination of supportive care and close monitoring for any changes in symptoms. The patient's clinical condition improved over a five-day period. His headache, unsteadiness, and fatigue completely resolved, and repeat CSF showed improvement. Inflammatory markers returned to normal, and the patient was discharged home.

## Discussion

The presented case of recurrent aseptic meningitis posed a significant diagnostic challenge due to the presence of multiple comorbidities. The absence of classic meningitis symptoms, such as fever and photophobia, combined with negative microbiological findings, further complicated the clinical assessment. Recurrent aseptic meningitis requires a thorough and systematic diagnostic approach, with a broad differential diagnosis encompassing both infectious and non-infectious etiologies, including viral infections, autoimmune disorders, and paraneoplastic syndromes [9]. Given the patient's clinical presentation, alternative diagnoses such as Mollaret's meningitis and autoimmune etiologies were considered. Mollaret's meningitis, a benign recurrent form of aseptic meningitis, often presents with negative CSF PCR results for HSV. There were reports of Mollaret's meningitis caused by other virus infections, including varicella-zoster virus (VZV), West Nile virus, EBV, human herpesvirus-6 (HHV-6), influenza virus, and enterovirus [10]. Additionally, reports underscore the atypical presentation patterns and the diagnostic utility of CSF cytology in identifying this rare condition [11]. However, in this case, a comprehensive viral panel and CSF cytology did not reveal any abnormalities. Further investigations targeted atypical fastidious bacteria, parasites, fungi, as well as non-infectious causes. Despite multiple CSF analyses, advanced imaging studies, and extensive microbiological, immunological, and paraneoplastic evaluations, no definitive etiology was identified. This exhaustive diagnostic process ultimately led to a diagnosis of idiopathic aseptic meningitis. Advancements in diagnostic techniques, including PCR, next-generation sequencing, and enhanced identification of autoimmune and paraneoplastic syndromes, have significantly improved the detection of causative agents in recurrent aseptic meningitis. Nonetheless, up to two-thirds of cases remain idiopathic [8]. Some studies suggest that mutations resulting in glycosylphosphatidylinositol (GPI) anchoring protein deficiency could represent a novel pathogenic mechanism for recurrent meningitis of unknown origin. The absence of these proteins may lead to excessive complement system activation, triggering recurrent meningeal inflammation and related symptoms [12]. However, in our case, we did not identify any low complement levels. Management of aseptic meningitis cases requires careful evaluation of clinical presentations and diagnostic findings. Most patients benefit from symptomatic treatment alone. Indomethacin, for example, is thought to reduce fever and inflammation by inhibiting periodic eicosanoid acid abnormalities in the brain. In contrast, treatments such as steroids, colchicine, antihistamines, and butylamine have shown limited efficacy [13,14]. There is currently no consensus regarding the necessity for long-term pharmacological therapy or recommendations for preventive treatment. In this case, the decision to initiate antiviral therapy despite negative viral panels underscores the importance of clinical judgment amid diagnostic uncertainty. The de‑escalation after specialist review illustrates the importance of avoiding unnecessary antimicrobials and minimizing potential side effects. Effective management of this patient involved multidisciplinary collaboration among neurology, infectious disease, and internal medicine teams, which was pivotal in formulating a comprehensive treatment plan. This case emphasizes the value of a multidisciplinary approach when addressing complex cases of recurrent aseptic meningitis. Clinicians must maintain a high index of suspicion, consider a wide range of differential diagnoses, and carefully balance the risks and benefits of therapeutic interventions.

## Conclusions

This case illustrates the complexities involved in diagnosing and managing recurrent aseptic meningitis, particularly in the absence of identifiable pathogens. A systematic, multidisciplinary strategy, coupled with vigilant monitoring, is essential for optimizing patient outcomes. Key insights include the necessity of considering viral etiologies in aseptic meningitis and acknowledging the diagnostic challenges posed by coexisting medical conditions. Further research into both infectious and non-infectious causes of recurrent meningitis is crucial for enhancing diagnostic accuracy and improving patient care.
